# Cost distribution and Monte Carlo simulation of financial risk under DRG-based prospective payment for laparoscopic cholecystectomy in patients with gallbladder polyps: a single-center retrospective study

**DOI:** 10.3389/fpubh.2026.1974837

**Published:** 2026-09-25

**Authors:** KeCheng Li, XiaoChan Xiang, Weiwei Zeng, Ge Li, JianWu Shi, XiaoLi Gao, QianWen Ye, Qi Xu, Wei Chen, ZhangChen Wu

**Affiliations:** Quality Control Office, Ruian People’s Hospital, Wenzhou, Zhejiang, China

**Keywords:** diagnosis-related groups, gallbladder polyp, gamma distribution, healthcare financing, hospitalization cost, laparoscopic cholecystectomy, Monte Carlo simulation

## Abstract

**Background:**

Under China’s Diagnosis-Related Group (DRG) prospective payment system, hospitals bear the financial risk of costs that exceed the fixed reimbursement standard, making case-level cost distribution and the probability of loss critical to departmental sustainability for high-volume, low-complexity procedures such as laparoscopic cholecystectomy.

**Objective:**

However, the probability distribution of hospitalization costs and the quantitative financial risk faced by a single institution operating under a fixed DRG payment standard for the group HS10B (laparoscopic cholecystectomy without common bile duct exploration) remain unclear.

**Methods:**

This retrospective single-center study included 475 consecutive patients who underwent laparoscopic cholecystectomy (surgery code 51.2300) for gallbladder polyps (DRG group HS10B) over a five-year period (2020–2024; annual case volume ranged from 50 to 136). Total hospitalization cost, medication cost, and material cost were extracted; other cost was derived as total cost minus medication cost minus material cost. Distributional properties were characterized using Kolmogorov–Smirnov normality testing and comparative fitting of normal, log-normal, and Gamma distributions (Akaike information criterion [AIC] and goodness-of-fit testing). Monte Carlo simulation (20,000 iterations), combining parametric resampling from the fitted Gamma distribution and non-parametric bootstrap resampling of the observed cohort, was used to estimate the probability distribution of case-level and aggregate financial margin relative to the local DRG reimbursement standard of ¥12,592.

**Results:**

Mean total hospitalization cost was ¥10,293.17 (SD 1,442.22; median ¥10,263.40, IQR ¥9,258.10–11,174.65), which was right-skewed (skewness 0.54) and best represented by a Gamma distribution (Kolmogorov–Smirnov *p* = 0.819). Twenty-two of 475 cases (4.63%) incurred costs exceeding the ¥12,592 breakeven threshold. Monte Carlo simulation estimated a mean per-case margin of ¥2,302.55 (95% simulation interval, −¥686.76 to ¥4,928.44) and a case-level loss probability of 6.2%. Bootstrap resampling of the observed cohort (*n* = 475) projected a mean aggregate margin of ¥1,091,888 (95% CI ¥1,029,920–1,153,715), with no net cohort-level loss observed among the 20,000 simulated resamples (upper one-sided 95% bound, 0.015%); an annual projection assuming 95 cases/year yielded a mean annual margin of ¥218,354 (95% CI ¥190,780–245,908), again with no simulated annual loss (upper bound, 0.015%); results were consistent when total cost was modeled as log-normal instead of Gamma. Total cost differed significantly across admission year (Kruskal–Wallis *p* < 0.001), declining from a mean of ¥10,172 in 2020 to ¥9,329 in 2024, with the empirical case-level loss rate falling correspondingly from 7.4 to 0% over the same interval.

**Conclusion:**

Inpatient cost for HS10B laparoscopic cholecystectomy follows a right-skewed distribution adequately described by a Gamma model; in this single-center cohort, case-specific cost volatility at the individual level was accompanied by a positive aggregate margin at the departmental level, consistent with a risk-pooling effect at this case volume. Hospital administrators should transition from conventional mean-centric accounting toward distribution-aware financial surveillance to better anticipate tail risk under prospective payment systems.

## Introduction

Gallbladder polyps are mucosal projections into the gallbladder lumen, incidentally identified on ultrasonography in 1.3–9.5% of the general population, and constitute an increasingly common indication for laparoscopic cholecystectomy as screening ultrasonography becomes more widely available (1). Although the great majority of resected polyps are benign cholesterol or pseudopolyps, current guidelines recommend cholecystectomy for polyps exceeding 10 mm, or smaller polyps accompanied by risk features such as age over 50 years, sessile morphology, or coexisting gallstones, reflecting the small but clinically important malignant potential of true adenomatous lesions (2–4). As a result, laparoscopic cholecystectomy for gallbladder polyps has become one of the highest-volume elective general-surgical procedures performed in tertiary and secondary hospitals.

Since 2019, China’s national medical insurance authority has progressively expanded Diagnosis-Related Group (DRG)-based prospective payment, under which hospitals receive a fixed, procedure-specific reimbursement irrespective of the actual resources consumed; laparoscopic cholecystectomy was incorporated into bundled DRG payment as this reform matured (5). Empirical evaluations across Chinese tertiary hospitals indicate that DRG-based payment is associated with reduced and more concentrated inpatient costs, particularly for medication and material expenditure, and with a narrower distribution of high-cost outliers relative to fee-for-service financing (5, 6). At the same time, because any expenditure above the fixed reimbursement standard is absorbed by the treating institution rather than the insurer, DRG payment transfers case-level financial risk from payer to provider, and this risk is a direct function of the shape of the underlying cost distribution rather than of the mean cost alone (7, 8).

Cost data for short-stay elective procedures such as laparoscopic cholecystectomy are characteristically right-skewed, reflecting a majority of routine, low-cost cases together with a smaller subset of complicated or resource-intensive admissions; simple comparison of group means therefore provides limited insight into the probability that an individual case, or an entire annual cohort, will exceed the DRG reimbursement ceiling. Although several studies have documented mean or median cost differences before and after DRG implementation for a range of Chinese surgical DRG groups (9, 10), traditional mean-centric accounting frequently fails to capture the fiscal tail risks posed by resource-intensive outliers. Therefore, quantitative, distribution-based evaluations of financial risk under a fixed local reimbursement standard remain a critical knowledge gap for the high-volume HS10B DRG group.

Therefore, the present study aimed to characterize the probability distribution of total hospitalization cost and its components (medication, material, and other costs) among patients undergoing laparoscopic cholecystectomy for gallbladder polyps under the HS10B DRG group, and to apply Monte Carlo simulation to quantify the case-level and aggregate financial margin relative to the local DRG reimbursement standard of ¥12,592, providing a distribution-based framework to inform departmental cost-containment and DRG contract-management decisions.

## Methods

### Study design and setting

This was a single-center, retrospective, descriptive cost-distribution study conducted using consecutive discharge records from the medical records and DRG grouping information system. All included admissions were classified under DRG group HS10B (laparoscopic cholecystectomy without common bile duct exploration; relative weight [RW] = 0.95) and were reimbursed against the corresponding local basic medical insurance DRG payment standard.

### Participants

Consecutive patients discharged between 2020 and 2024 with a principal diagnosis of gallbladder polyp (ICD-10 K82.802) who underwent laparoscopic cholecystectomy (surgery code 51.2300) and were grouped into DRG HS10B were eligible. Patients were included if the DRG grouping record, principal diagnosis, principal procedure, and total hospitalization cost were complete and internally consistent (total cost equal to the sum of its components). 475 admissions were included in the final analytic cohort. Annual admission counts (one late-December 2019 admission grouped with the 2020 cohort) were 54, 117, 136, 118, and 50 for 2020 through 2024, respectively, an annual case volume ranging from 50 to 136 around the study-period mean of 95.

### Data collection

For each admission, the following variables were extracted from the hospital information system: patient sex, age, DRG code and relative weight, principal diagnosis code and name, principal procedure code and name, discharge disposition, total hospitalization cost, total medication cost, and total medical material (consumables) cost. Other costs, a residual category plausibly encompassing examination, laboratory, bed-occupancy, nursing, and other service charges not separately itemized in the extracted dataset, were calculated for each admission record by subtracting medication costs and material costs from the total cost. This additive cost decomposition follows standard practice in Chinese hospital cost accounting studies (6, 11). Total hospitalization cost, as recorded in the hospital information system, corresponds to the discharge settlement charge for the admission, that is, the sum of the amount paid by the basic medical insurance fund and the amount paid out of pocket by the patient; it is therefore a billing-based (charge) measure rather than an internally audited hospital cost-accounting or economic-cost figure.

### Outcome variables

The primary outcome was total hospitalization cost per admission and its probability distribution. Secondary outcomes were the three cost components (medication, material, and other cost); the case-level payment–cost differential, defined as the local DRG-HS10B payment amount (¥12,592) minus the recorded total hospitalization cost for each case; the proportion of cases in which recorded total hospitalization cost exceeded the DRG payment amount (case-level loss); and the simulated aggregate (cohort- and annual-level) payment–cost differential obtained from Monte Carlo simulation. The ¥12,592 amount represented the fixed DRG payment actually disbursed to the hospital by the local basic medical insurance fund for an HS10B admission.

### Statistical analysis

For the primary cost variable (total hospitalization cost), three candidate parametric distributions (normal, log-normal, and Gamma) were fitted by maximum likelihood. Model performance was compared using the Akaike information criterion (AIC) and a distribution-specific Kolmogorov–Smirnov goodness-of-fit test. The Gamma distribution was selected as the primary model because it achieved the best absolute fit (highest KS *p* value) and is the conventional choice for right-skewed healthcare cost data; the log-normal distribution, which had a marginally lower AIC (ΔAIC = 1.9), was retained as a sensitivity comparator.

A Monte Carlo simulation (20,000 iterations) was then implemented at three levels: (i) case-level simulation, drawing single observations from the fitted Gamma distribution to estimate the distribution of per-case payment–cost differential and simulated loss probability; (ii) cohort-level non-parametric bootstrap, resampling with replacement 475 observations from the empirical cost distribution per iteration to estimate the 5-year aggregate differential; and (iii) annual-projection simulation, assuming a base case of 95 admissions/year (475 cases/5 years) drawn from the fitted Gamma distribution to estimate the projected annual departmental differential. The annual-projection simulation assumes that future case volume, reimbursement standard, patient case-mix, and cost structure remain comparable to those observed in 2020–2024. To evaluate sensitivity to case volume, the annual simulation was repeated at 20% lower and higher volumes (76 and 114 cases/year). Because a zero-event result in 20,000 iterations reflects simulation resolution rather than a true zero probability, an approximate upper one-sided 95% confidence bound was calculated using the rule-of-three approximation (3/n).

For comparative analyses, total hospitalization cost across admission years (2020–2024) was evaluated using the Kruskal–Wallis test to assess temporal trends. Between-sex comparisons of cost variables used the Mann–Whitney U test with rank-biserial correlation as the effect size, given non-normality; for this exploratory comparison across four cost categories, a Bonferroni-adjusted significance threshold of α = 0.0125 (0.05/4) was applied. Results are reported as simulation means with 95% simulation intervals (2.5th–97.5th percentiles). All statistical tests were two-sided with α = 0.05 unless adjusted. Analyses were performed in Python 3.11 using pandas 2.x, NumPy, SciPy 1.x, statsmodels, and Matplotlib.

## Results

A total of 475 patients undergoing laparoscopic cholecystectomy for gallbladder polyps under DRG group HS10B between 2020 and 2024 were analyzed. Most patients were female (289/475, 60.8%), and mean age was 48.35 ± 12.57 years (range 17–84 years); age was consistent with a normal distribution (Kolmogorov–Smirnov *p* = 0.549) (Table 1).

**Table 1:** Baseline characteristics and cost variables of the study cohort (*n* = 475).

Variable	Value
Sex, female/male, *n* (%)	289 (60.8%)/186 (39.2%)
Age, years, mean ± SD	48.35 ± 12.57
Age, years, range	17–84
Total cost, CNY, mean ± SD	10,293.17 ± 1,442.22
Total cost, CNY, median [IQR]	10,263.40 [9,258.10–11,174.65]
Total cost, CNY, range	6,513.80–16,703.40
Medication cost, CNY, mean ± SD	2,295.99 ± 695.03
Medication cost, CNY, median [IQR]	2,182.95 [1,837.33–2,643.51]
Material cost, CNY, mean ± SD	2,043.37 ± 813.05
Material cost, CNY, median [IQR]	2,114.91 [1,592.63–2,552.70]
Other cost, CNY, mean ± SD	5,953.82 ± 767.40
Other cost, CNY, median [IQR]	5,872.55 [5,404.08–6,293.51]
DRG relative weight (RW)	0.95 (all cases)

SD, standard deviation; IQR, interquartile range; CNY, Chinese yuan; DRG, Diagnosis-Related Group; RW, relative weight.

Mean total hospitalization cost was ¥10,293.17 ± 1,442.22 (median ¥10,263.40, IQR ¥9,258.10–11,174.65; range ¥6,513.80–16,703.40), with a positive skewness of 0.54, indicating a right-skewed distribution with a longer tail toward high-cost cases (Table 1; Figure 1a). Medication cost (mean ¥2,295.99 ± 695.03) and other cost (mean ¥5,953.82 ± 767.40) showed significant departure from normality (Kolmogorov–Smirnov *p* = 0.009 and *p* < 0.001, respectively), whereas material cost (mean ¥2,043.37 ± 813.05) did not significantly depart from normality (*p* = 0.118) (Table 1).

**Figure 1:**
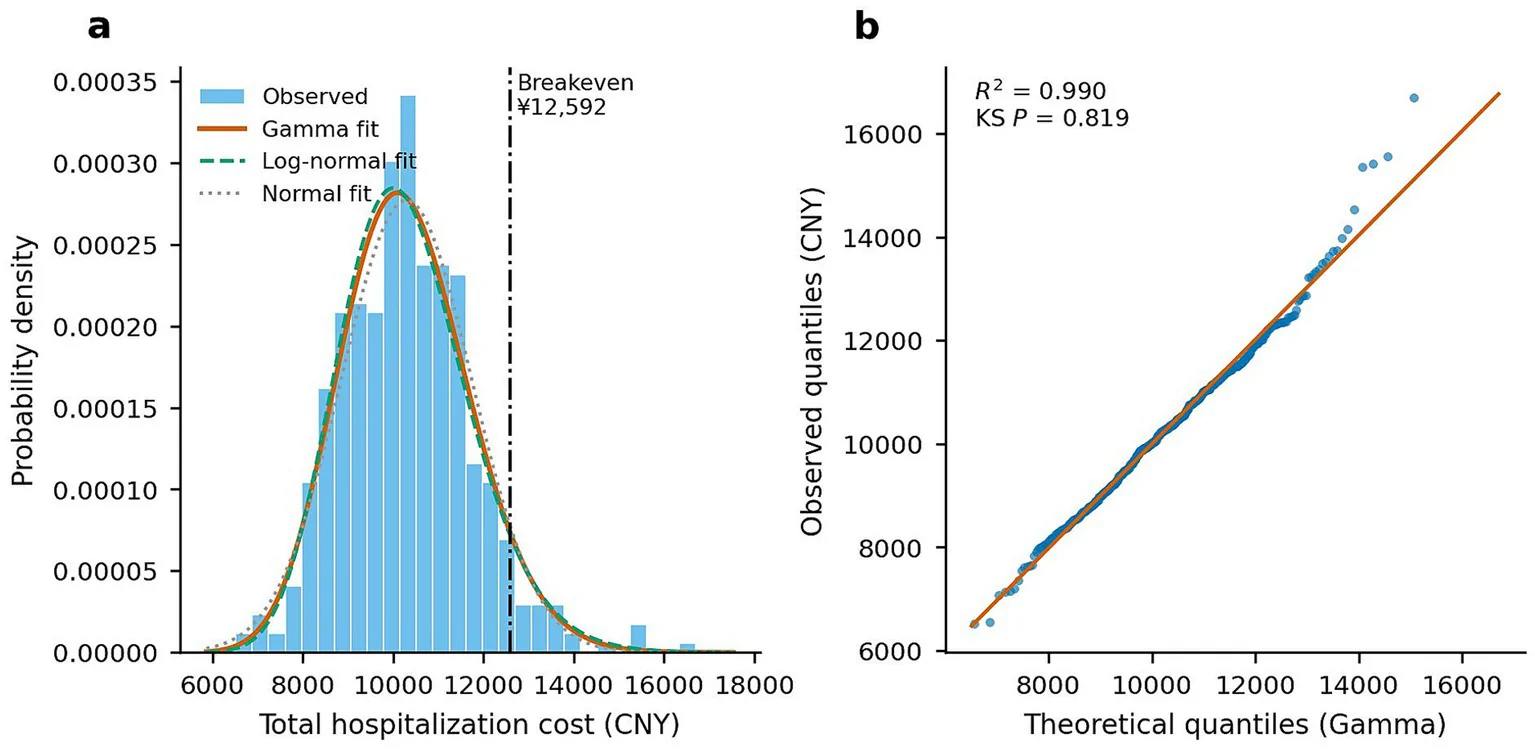
Distribution of total hospitalization cost and goodness-of-fit assessment. **(a)** Histogram of observed total hospitalization cost (*n* = 475) with overlaid Gamma, log-normal, and normal probability density fits; the dash-dot vertical line indicates the local DRG-HS10B breakeven reimbursement standard (¥12,592). **(b)** Gamma quantile–quantile (Q–Q) plot comparing observed cost quantiles against theoretical Gamma quantiles; the diagonal line indicates the fitted relationship. *R*^2^ and Kolmogorov–Smirnov (KS) *p* value for the Gamma fit are annotated.

Among the three candidate parametric models for total cost, the log-normal distribution achieved the lowest AIC (8,244.7), followed by the Gamma distribution (AIC 8,246.6) and the normal distribution (AIC 8,261.2); however, the Gamma distribution achieved the best absolute goodness of fit on Kolmogorov–Smirnov testing (smallest KS distance, D = 0.029; *p* = 0.819, versus *p* = 0.526 for log-normal and *p* = 0.574 for normal) (Table 2, Figures 1a,b). Although the log-normal model achieved marginally lower AIC (ΔAIC = 1.9), the Gamma distribution was selected for simulation because it provides the closest absolute fit to the observed data and is a standard choice for right-skewed positive continuous variables in healthcare cost modeling (7, 12). Both models yield substantively identical simulation results. The Gamma distribution (shape a = 51.96, scale = 198.09; implied mean = ¥10,293.2, matching the empirical mean) was therefore adopted as the primary model for Monte Carlo simulation. As a sensitivity analysis, the case-level and annual-projection simulations were repeated using the fitted log-normal distribution in place of Gamma; results were closely comparable across the two models (case-level loss probability 6.6% versus 6.2%; mean annual margin ¥218,572 versus ¥218,354; Table 3), supporting the conclusion that the choice between these two similarly well-fitting distributions did not materially affect the simulation-based findings.

**Table 2:** Distribution fitting for total hospitalization cost.

Distribution	Parameters	AIC	KS statistic	KS *p*
Normal	μ = 10,293.17; σ = 1,440.70	8,261.2	0.036	0.574
Log-normal	s = 0.139; scale = 10,194.29	8,244.7	0.037	0.526
Gamma*	a = 51.96; scale = 198.09	8,246.6	0.029	0.819

^*^Gamma distribution selected as the final model for Monte Carlo simulation on the basis of the smallest KS distance and best absolute Kolmogorov–Smirnov (KS) goodness-of-fit p value and conventional use for right-skewed healthcare cost data; log-normal, which achieved marginally lower AIC, was retained as a sensitivity comparator. AIC, Akaike information criterion.

**Table 3:** Monte Carlo simulation results (20,000 iterations; breakeven = ¥12,592).

Simulation level	Mean margin	95% simulation interval/CI	P(loss)
Case-level (Gamma, parametric)	¥2,302.55/case	−¥686.76 to ¥4,928.44	6.2%
Empirical (observed cohort)	¥2,298.83/case	—	4.63% (22/475)
Cohort aggregate (bootstrap, *n* = 475)	¥1,091,888	¥1,029,920 to ¥1,153,715	0/20,000 (≤0.015%)
Annual projection (*n* = 95/year, Gamma)	¥218,354	¥190,780 to ¥245,908	0/20,000 (≤0.015%)

P(loss) denotes the simulated probability that cost/margin falls on the loss side of the ¥12,592 breakeven threshold (case-level) or that aggregate margin is negative (cohort/annual level). 0/20,000 values indicate that no loss event was observed among the 20,000 iterations; the corresponding approximate upper one-sided 95% bound (rule-of-three approximation, 3/20,000) is 0.015%, not a true probability of zero. Sensitivity analysis using the log-normal distribution in place of Gamma yielded closely comparable case-level (P(loss) 6.6%) and annual-level (mean margin ¥218,572; 0/20,000 loss events) results. Repeating the annual simulation at 76 and 114 cases/year (±20% of the base case-volume assumption) yielded mean annual margins of ¥174,777 and ¥262,169, respectively, with 0/20,000 loss events at either volume. CI, confidence interval.

Other cost was the largest component of total cost, accounting for 57.8% of the mean total, followed by medication cost (22.3%) and material cost (19.9%) (Figures 2a,b). On univariate comparison by sex, material cost was higher at the nominal *p* < 0.05 threshold in female than male patients (median ¥2,158.76 [IQR ¥1,615.90–2,606.63] versus ¥1,908.06 [IQR ¥1,396.08–2,502.30]; Mann–Whitney U = 30,461, *p* = 0.014, *r* = −0.133), while total cost (*p* = 0.052), medication cost (*p* = 0.062), and other cost (*p* = 0.621) did not differ significantly between sexes (Table 4). This sex comparison was exploratory rather than a prespecified primary analysis; after applying a Bonferroni-corrected threshold for the four cost comparisons (α = 0.0125), the material-cost difference no longer met statistical significance (*p* = 0.014).

**Figure 2:**
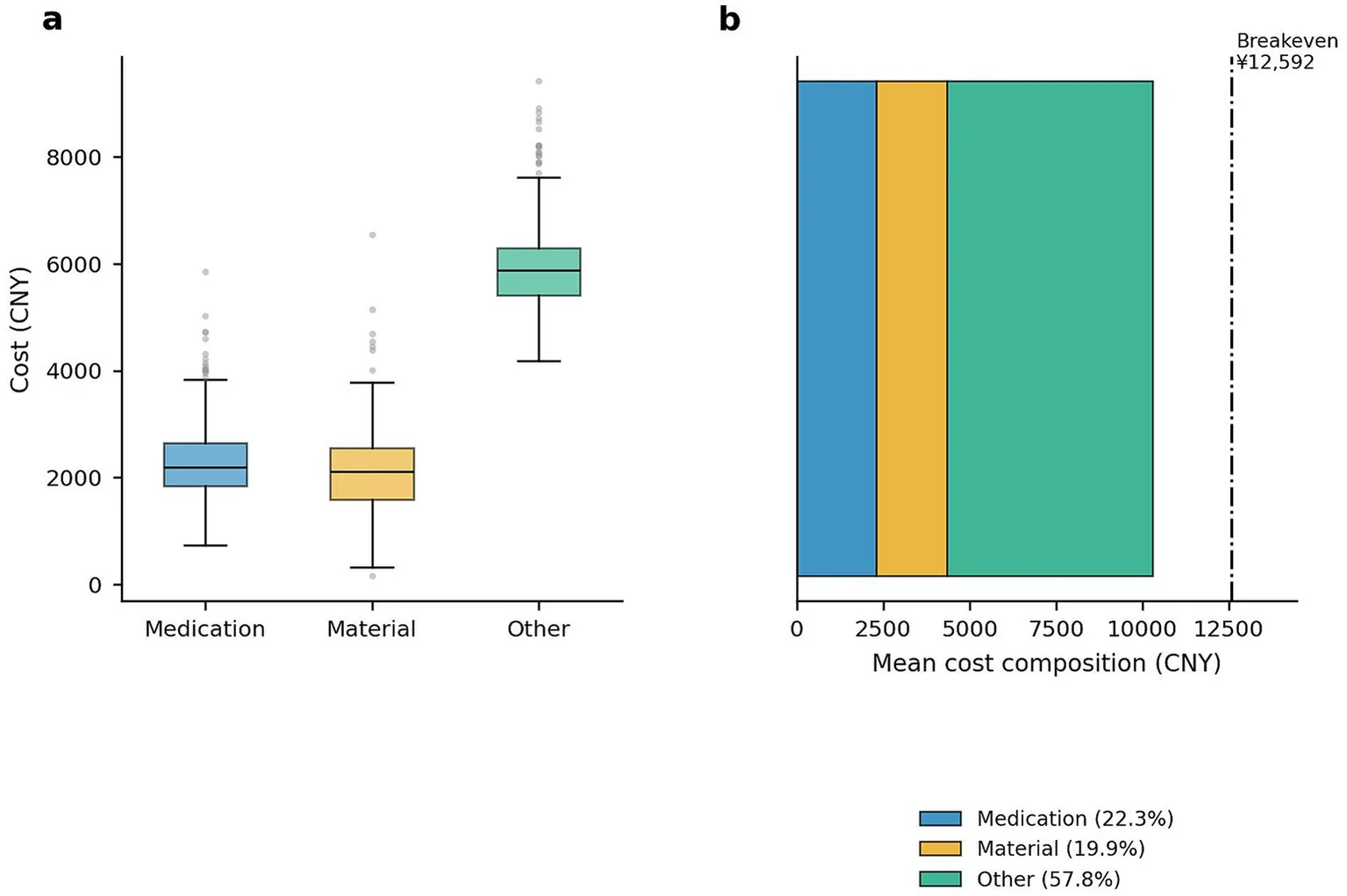
Cost composition of laparoscopic cholecystectomy hospitalizations. **(a)** Box plots showing the distribution of medication, material, and other cost (*n* = 475); boxes represent the interquartile range (IQR) with the median indicated by the central line, whiskers extend to 1.5 × IQR, and outliers are shown as individual points. **(b)** Mean cost composition shown as a stacked horizontal bar, with the percentage contribution of each component to mean total cost; the dash-dot vertical line indicates the ¥12,592 breakeven reimbursement standard.

**Table 4:** Cost comparison by patient sex.

Cost variable	Female, median [IQR]	Male, median [IQR]	U	*p*	*r*
Total cost	10,352.50 [9,461.20–11,267.00]	10,076.55 [9,083.80–11,029.05]	29,712	0.052	−0.105
Medication cost	2,216.27 [1,871.24–2,708.30]	2,141.95 [1,784.36–2,569.95]	29,601	0.062	−0.101
Material cost	2,158.76 [1,615.90–2,606.63]	1,908.06 [1,396.08–2,502.30]	30,461	0.014	−0.133
Other cost	5,855.10 [5,402.97–6,259.33]	5,910.91 [5,407.86–6,312.52]	26,154	0.621	0.027

Mann–Whitney U test; *r*, rank-biserial correlation (effect size). This sex comparison is exploratory; at the nominal threshold (*p* < 0.05) material cost differed by sex, but this did not survive a Bonferroni-corrected threshold (α = 0.0125) for the four comparisons shown. IQR, interquartile range.

Against the local DRG-HS10B reimbursement standard of ¥12,592, 22 of 475 cases (4.63%) had actual total cost exceeding the breakeven threshold, i.e., an empirical case-level loss rate of 4.63% (95% CI 2.6–7.7%, Wilson score method). Monte Carlo simulation from the fitted Gamma distribution (20,000 iterations) estimated a mean simulated per-case cost of ¥10,289.45 and a mean per-case financial margin of ¥2,302.55 (95% simulation interval, −¥686.76 to ¥4,928.44), with a simulated case-level loss probability of 6.2%, modestly higher than the observed empirical rate, consistent with the additional right-tail probability mass captured by the fitted distribution beyond the observed sample range (Figure 3a; Table 3).

**Figure 3:**
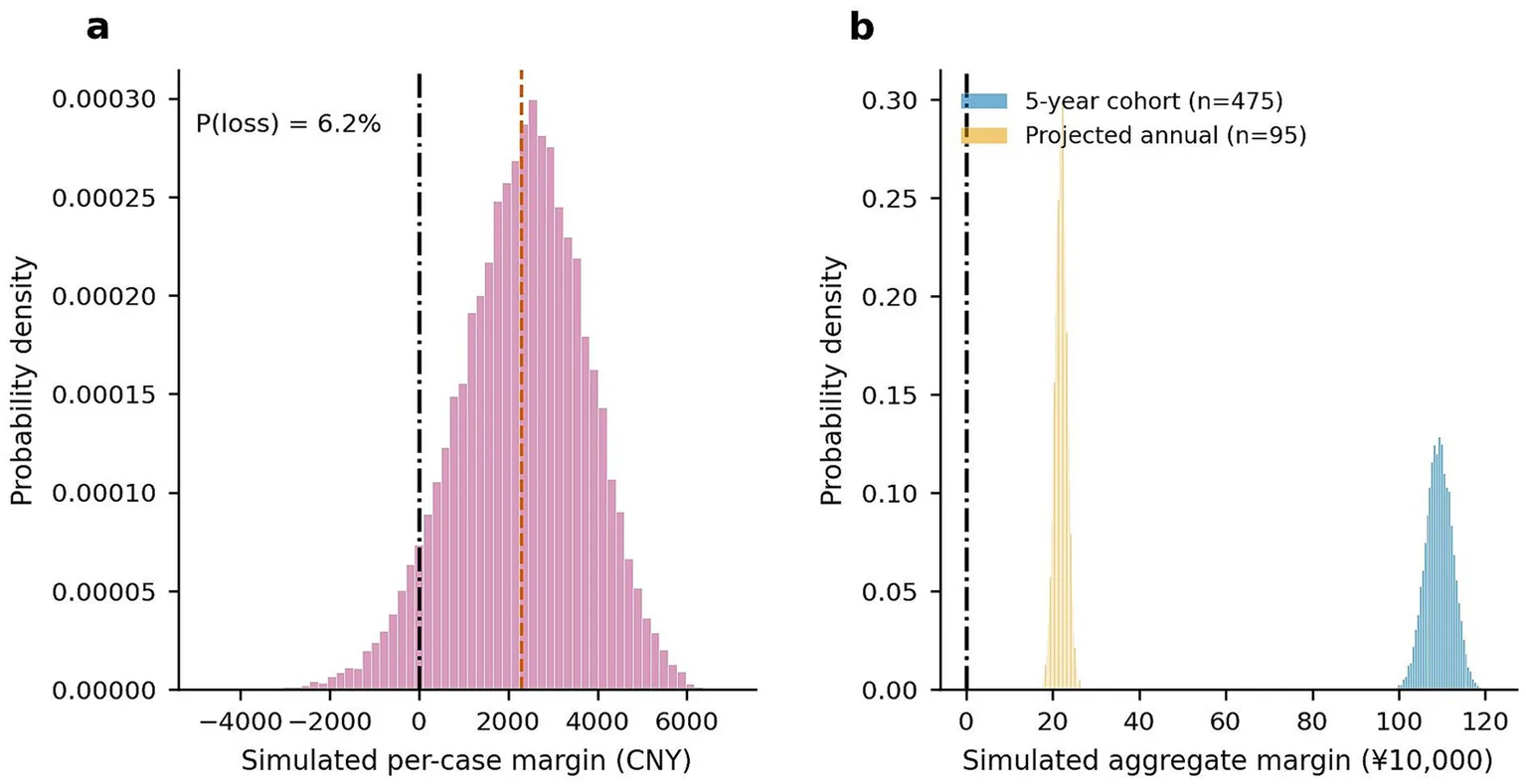
Monte Carlo simulation of case-level and aggregate financial margin. **(a)** Simulated distribution of per-case financial margin (reimbursement standard − simulated cost) from 20,000 Gamma-distribution draws; the dash-dot vertical line indicates the breakeven margin of ¥0, the dashed line indicates the simulation mean, and the simulated probability of case-level loss, P(loss), is annotated. **(b)** Simulated distribution of aggregate financial margin for the observed 5-year cohort (non-parametric bootstrap, *n* = 475 per iteration) and for a projected annual cohort (parametric Gamma simulation, *n* = 95 per iteration), both expressed in units of ¥10,000; note that the y-axis label in the figure uses (ten thousand yuan) as the unit: the dash-dot vertical line indicates zero net margin.

Non-parametric bootstrap resampling of the observed 475-case cohort (20,000 iterations) estimated a mean aggregate 5-year margin of ¥1,091,888 (95% CI ¥1,029,920–1,153,715; SD ¥31,586); no net cohort-level loss was observed among the 20,000 simulated resamples (upper one-sided 95% bound, 0.015%, rule-of-three approximation), and the mean simulated case-level loss rate was 4.63%, closely matching the empirical value. Annual-projection simulation, assuming an average annual case volume of 95 (475 cases / 5 years) drawn from the fitted Gamma distribution, estimated a mean annual departmental margin of ¥218,354 (95% CI ¥190,780–245,908; SD ¥13,965); again, no simulated annual net loss was observed (upper bound, 0.015%) (Figure 3b; Table 3). Repeating the annual simulation at case volumes 20% below and above the base assumption (76 and 114 cases/year) yielded proportionally scaled mean annual margins of ¥174,777 and ¥262,169, respectively, with no simulated net loss at either volume, indicating that the estimated annual margin remained positive across these three case-volume scenarios.

Total hospitalization cost differed significantly across admission years (Kruskal–Wallis H = 43.1, *p* < 0.001). Mean total cost was ¥10,172 in 2020, ¥10,772 in 2021, ¥10,453 in 2022, ¥10,098 in 2023, and ¥9,329 in 2024. Applying the current ¥12,592 reimbursement threshold uniformly across all study years, the empirical case-level loss rate declined correspondingly from 7.4% (4/54) in 2020 to 6.8% (8/117) in 2021, 4.4% (6/136) in 2022, 3.4% (4/118) in 2023, and 0.0% (0/50) in 2024.

## Discussion

In this single-center retrospective analysis of 475 patients undergoing laparoscopic cholecystectomy for gallbladder polyps under DRG group HS10B, total hospitalization cost was right-skewed and best modeled by a Gamma distribution, 4.63% of cases exceeded the local DRG reimbursement standard of ¥12,592, and Monte Carlo simulation indicated that although individual cases carried a 4.6–6.2% probability of financial loss, the aggregate 5-year cohort and projected annual departmental margins were consistently positive, with no simulated net loss observed at either level (upper one-sided 95% bound, 0.015%). The higher simulated case-level loss probability (6.2%) relative to the empirical rate (4.63%) reflects the fact that the fitted Gamma distribution extrapolates a small amount of additional right-tail probability mass beyond the specific 475 cases observed; the two figures describe, respectively, a model-based estimate of the underlying risk and the realized frequency within this particular cohort.

This empirical pattern—where a non-trivial case-level loss risk coexists with a positive simulated aggregate margin—is compatible with statistical risk pooling across cases: losses generated by individual high-cost outliers in the right-tail of the Gamma distribution were offset in the aggregate simulations by the larger number of lower-cost cases. These findings suggest that, for a relatively standardized and low-complexity DRG group such as HS10B, higher case volume may attenuate the aggregate impact of individual high-cost outliers, though this single-center observation should not be generalized to departmental sustainability under DRG regimes more broadly. Because total cost was right-skewed with a coefficient of variation of only 0.14, the great majority of cases clustered well below the reimbursement ceiling, while a small right-tail subset of more resource-intensive admissions absorbed cost overruns without materially altering the group mean. This distributional structure explains why per-case risk under DRG payment cannot be adequately characterized by comparing group means alone, and supports the use of full-distribution and simulation-based approaches, as recommended in prior work on the concentration and dispersion of DRG-era inpatient costs, when institutions evaluate the financial sustainability of a given DRG contract (6, 13).

The dominance of “other cost” (57.8% of the mean total, encompassing bed, nursing, examination, and service fees not separately itemized) over medication and material cost together is consistent with the broader cost-containment effect that has been attributed to DRG-based payment reform in China, under which fixed reimbursement is thought to incentivize protocol-driven reductions in discretionary medication and consumable use while core service and bed-day charges remain comparatively fixed (6, 13). However, other cost was calculated as a residual (total cost minus medication and material cost) rather than extracted as itemized bed, nursing, examination, and service charges; the dataset therefore cannot establish which of these underlying components drove the observed residual, and the interpretation above should be read as a plausible composition rather than a component-level finding. The modestly higher material cost observed in female patients may be associated with differences in surgical consumable selection or comorbidity profile between sexes, but this association should not be interpreted as a direct causal driver of cost variation given the observational design, the exploratory nature of this comparison, and the absence of adjustment for other clinical covariates in the present dataset; indeed, this difference did not survive Bonferroni correction for multiple comparisons. The marginal AIC advantage of the log-normal model (ΔAIC = 1.9, indicating that the two models had similar support from the AIC criterion (14)) was outweighed by the Gamma distribution’s superior KS goodness-of-fit (*p* = 0.819 versus 0.526) and its convention as the canonical distribution for right-skewed healthcare cost data in generalized linear models (12). Both distributions yield nearly identical simulation results, and our conclusions are robust to this choice.

These findings are broadly consistent with prior evaluations of DRG-based payment in Chinese tertiary hospitals, which have reported that DRG reimbursement is associated with a more concentrated inpatient cost distribution and a reduced proportion of high-cost outlier cases relative to fee-for-service financing, and that cost containment under global-budget DRG payment models has been observed across a range of surgical and medical DRG groups (5, 6, 13). The present study extends this literature by providing an explicit, simulation-based quantification of case-level and departmental loss probability for a single, high-volume, low-complexity DRG group, complementing the largely mean-comparison-based evidence reported previously (5, 6, 13).

This study has several limitations. First, as a single-center, single-DRG retrospective analysis lacking systematic data on comorbidity burden, surgical complexity, or intraoperative complications, our findings may not fully generalize to other institutions, and the clinical determinants driving the 22 high-cost cases exceeding the reimbursement threshold could not be characterized. Second, because all included patients had regular physician-advised discharges, cost variations related to adverse outcomes or mortality were not evaluated. Third, the Monte Carlo projections assume structural stability in future case-mix, unit costs, clinical pathways, and reimbursement rules; they should thus be interpreted as scenario analyses rather than dynamic forecasts. Fourth, total hospitalization cost was defined by billed settlement charges (combining insurance disbursements and patient copayments) rather than internally audited economic costs. While the ¥12,592 reimbursement is confirmed as the amount disbursed to the hospital by the insurance fund, unitemized patient out-of-pocket costs preclude reconstructing the institution’s full net accounting margin. Fifth, because historical year-specific reimbursement standards were unavailable, applying the current ¥12,592 benchmark uniformly across the 2020–2024 cohort may misestimate historical loss rates during periods of secular cost shifts and centralized procurement reforms. Nevertheless, this study provides a reproducible, distribution-aware simulation pipeline that administrators can recalibrate dynamically as real-time pricing evolves. Finally, these estimates may not generalize to laparoscopic cholecystectomy performed in clinically complex or concomitant operative settings. For instance, a large MBSAQIP analysis of over 1 million bariatric operations showed that concomitant laparoscopic cholecystectomy was associated with modest increases in select postoperative complications despite a favorable short-term safety profile, underscoring how surgical context influences resource utilization and outcomes (15).

From a departmental management perspective, these findings suggest that routine monitoring of the full cost distribution—rather than the mean cost alone—together with periodic Monte Carlo re-simulation as case-mix or unit costs evolve, may help institutions anticipate and manage case-level financial risk under DRG-based prospective payment for high-volume elective procedures such as laparoscopic cholecystectomy.

## Conclusion

This study demonstrates that the inpatient cost profile of laparoscopic cholecystectomy under the DRG-HS10B group follows a right-skewed distribution adequately described by a Gamma model. This cost structure generates a tangible but low probability of per-case financial loss. Crucially, our simulation framework reveals an empirical decoupling between micro-level variance and macro-level fiscal sustainability: while individual resource-intensive outliers occasionally breach the prospective reimbursement ceiling, the aggregate departmental margin remained positive under the specified simulation assumptions, with no net-loss events observed among 20,000 simulated iterations (upper one-sided 95% bound, 0.015%).

From a healthcare management perspective, these findings advocate for a paradigm shift in DRG financial surveillance—moving beyond static, mean-centric accounting toward distribution-aware risk monitoring. The integration of parametric Gamma modeling and non-parametric bootstrap simulations may support distribution-aware monitoring of DRG-related financial risk for clinical departments and hospital administrators, complementing rather than replacing conventional mean-based cost review.

## Data Availability

The raw data supporting the conclusions of this article will be made available by the authors, without undue reservation.
